# Signalling pathway involved in nitric oxide synthase type II activation in chondrocytes: synergistic effect of leptin with interleukin-1

**DOI:** 10.1186/ar1708

**Published:** 2005-03-04

**Authors:** Miguel Otero, Rocío Lago, Francisca Lago, Juan Jesús Gomez Reino, Oreste Gualillo

**Affiliations:** 1 NEIRID (NeuroEndocrine Interactions in Rheumatology and Inflammatory Diseases) Laboratory, Santiago University Clinical Hospital, Research Laboratory 4, Santiago de Compostela, Spain; 2 Laboratory of Molecular and Cellular Cardiology, Santiago University Clinical Hospital, Research Laboratory 1, Santiago de Compostela, Spain; 3 Rheumatology Division, Santiago University Clinical Hospital, Santiago de Compostela, Spain; 4 Department of Medicine, School of Medicine, University of Santiago de Compostela, Santiago de Compostela, Spain

## Abstract

The objective of the present study was to investigate the effect of leptin, alone or in combination with IL-1, on nitric oxide synthase (NOS) type II activity *in vitro *in human primary chondrocytes, in the mouse chondrogenic ATDC5 cell line, and in mature and hypertrophic ATDC5 differentiated chondrocytes. For completeness, we also investigated the signalling pathway of the putative synergism between leptin and IL-1. For this purpose, nitric oxide production was evaluated using the Griess colorimetric reaction in culture medium of cells stimulated over 48 hours with leptin (800 nmol/l) and IL-1 (0.025 ng/ml), alone or combined. Specific pharmacological inhibitors of NOS type II (aminoguanidine [1 mmol/l]), janus kinase (JAK)2 (tyrphostin AG490 and Tkip), phosphatidylinositol 3-kinase (PI3K; wortmannin [1, 2.5, 5 and 10 μmol/l] and LY294002 [1, 2.5, 5 and 10 μmol/l]), mitogen-activated protein kinase kinase (MEK)1 (PD098059 [1, 5, 10, 20 and 30 μmol/l]) and p38 kinase (SB203580 [1, 5, 10, 20 and 30 μmol/l]) were added 1 hour before stimulation. Nitric oxide synthase type II mRNA expression in ATDC5 chondrocytes was investigated by real-time PCR and NOS II protein expression was analyzed by western blot. Our results indicate that stimulation of chondrocytes with IL-1 results in dose-dependent nitric oxide production. In contrast, leptin alone was unable to induce nitric oxide production or expression of NOS type II mRNA or its protein. However, co-stimulation with leptin and IL-1 resulted in a net increase in nitric oxide concentration over IL-1 challenge that was eliminated by pretreatment with the NOS II specific inhibitor aminoguanidine. Pretreatment with tyrphostin AG490 and Tkip (a SOCS-1 mimetic peptide that inhibits JAK2) blocked nitric oxide production induced by leptin/IL-1. Finally, wortmannin, LY294002, PD098059 and SB203580 significantly decreased nitric oxide production. These findings were confirmed in mature and hypertrophic ATDC5 chondrocytes, and in human primary chondrocytes. This study indicates that leptin plays a proinflammatory role, in synergy with IL-1, by inducing NOS type II through a signalling pathway that involves JAK2, PI3K, MEK-1 and p38 kinase.

## Introduction

Chondrocytes are the predominant cells in mature cartilage that synthesize and maintain the integrity of cartilage-specific extracellular matrix. In rheumatoid arthritis and osteoarthritis the phenotype of chondrocytes changes, and apoptosis and extracellular matrix degradation occur [1-3]. These severe perturbations in cartilage homeostasis may be mediated in part by nitric oxide (NO). This gaseous mediator is induced by several proinflammatory cytokines, including IL-1.

Leptin, the *OB *gene product, is a 16 kDa hormone that is synthesized by adipocytes. Leptin regulates food intake and energy expenditure, but it also modulates neuroendrocrine function [4]. It is involved in immune modulation in that it influences the innate immune response by promoting activation of monocyte/macrophages, chemotaxis and activation of neutrophils, and activation of natural killer cells [5]. Furthermore, leptin influences adaptive immunity by increasing the expression of adhesion molecules by CD4^+ ^T cells, and promoting proliferation and secretion of IL-2 by naïve CD4^+ ^T cells [5-7]. Leptin has also been found to influence bone growth [8] and inflammation [9].

High leptin levels are associated with obesity, which is a risk factor for osteoarthritis [10-12]. Interestingly, in patients with osteoarthritis leptin is present in synovial fluid and is expressed by articular chondrocytes [13], and normal human chondrocytes express the functional Ob-Rb leptin receptor isoform [14]. It is unlikely that leptin alone acts on cartilage to trigger an inflammatory response; rather, it may associate with other proinflammatory cytokines to amplify inflammation and enhance damage to cartilage. We recently demonstrated a synergistic effect of leptin with IFN-γ on nitric oxide synthase (NOS) type II activity in cultured chondrocytes that was mediated by the janus kinase (JAK)2 [15]. In the present study we investigated whether leptin synergizes with IL-1, an abundant mediator of inflammation and cartilage destruction [16,17], to activate NOS type II in chondrocytes. To gain further insights into the mechanism of action of this putative synergism, we also analyzed the role played by several intracellular kinases by using specific pharmacological inhibitors.

## Materials and methods

### Reagents

Foetal bovine serum, tissue culture media, media supplements, mouse and human recombinant leptin, mouse recombinant IL-1, tyrphostin AG490, wortmannin, LY294002, PD098059 and SB203580 were purchased from Sigma (St Louis, MO, USA) unless otherwise specified. RT-PCR reagents were purchased from Invitrogen (Carlsbad, CA, USA) and Stratagene (La Jolla, CA, USA). Tkip (WLVFFVIFYFFR), a suppressor of cytokine signalling (SOCS)-1 mimetic peptide that inhibits JAK2 autophosphorylation, was generously provided by Dr Howard M Johnson (Institute of Food and Agricultural Science, Department of Microbiology and Cell Science, University of Florida, Gainesville, FL, USA).

### Cell culture

The clonal chondrogenic cell line ATDC5 was chosen for these studies because it has been shown to be a useful *in vitro *model for examining the multistep differentiation of chondrocytes. Undifferentiated ATDC5 cells proliferate rapidly until they reach confluence, at which point they undergo growth arrest. When treated with insulin, transferrin and sodium selenite, confluent ATDC5 cells re-enter a proliferative phase and form cartilaginous matrix nodules (mature chondrocytes). As differentiation progresses, these cells undergo a late differentiation phase, becoming hypertrophic, calcifying chondrocytes that synthesize type X collagen and osteopontin – a marker of terminal chondrocyte differentiation [18]. ATDC5 cells were a kind gift from Dr Agamemnon E Grigoriadis (Department of Craniofacial Development, King's College, London Guy's Hospital, London, UK). Unless otherwise specified, cells were cultured in Dulbecco's modified Eagle's medium/Hams' F12 medium supplemented with 5% foetal bovine serum, 10 μg/ml human transferrin, 3 × 10^-8 ^mol/l sodium selenite and antibiotics (50 U/ml penicillin and 50 μg/ml streptomycin).

In some experiments, conducted to demonstrate that leptin/IL-1 synergism does not appear to depend on the differentiation state of the chondrocytes, chondrogenic ATDC5 cells were differentiated into mature and hypertrophic chondrocytes, as described by Thomas and coworkers [19]. Briefly, cells were plated at an initial density of 2 × 10^4 ^cells/well in 24-well plates. Cells were cultured in the above-mentioned medium supplemented with 10 μg/ml of human recombinant insulin (Novo Nordisk A/S, Bagsvaerd, Denmark). Culture was continued for a further 15 or 21 days, with replacement of medium every other day. As expected, ATDC5 cultures treated with insulin underwent progressive differentiation from 0 to 21 days as compared with untreated cultures. This differentiation was qualitatively characterized by increased formation of cartilage nodules and enhanced staining with alcian blue dye, which is indicative of cartilage proteoglycan accumulation.

In other experiments (data not shown), the differentiation from days 0 to 21 was further evidenced by sequential increases in type II collagen, aggrecan and type X collagen mRNAs. The early and mature chondrocyte marker type II collagen was expressed in undifferentiated ATDC5 cells; the level began to increase at day 3, peaked at days 7–10 and gradually declined after day 15. The expression profile of aggrecan mimicked that of type II collagen but with a slight delay of a couple of days. The decline in expression of both chondrocyte markers coincided with the onset of late-stage chondrocyte differentiation. The expression of the hypertrophic chondrocyte marker type X collagen began at days 12 and 13. The expression patterns of these early and late chondrocyte markers were consistent with previous findings in ATDC5 cells regarding *in vivo *chondrocyte differentiation. We do not illustrate findings regarding the differentiation of ATDC5 cells because they are extensively reported in literature [19].

### Cartilage harvest and human chondrocyte isolation

Human normal articular cartilage samples were obtained from knee joints of patients undergoing leg amputations from above the knee because of peripheral vascular disease. (Permission from the local ethical committee was granted.) None of the patients had a clinical history of arthritis or any other pathology affecting the cartilage, and the specimens appeared normal on morphological examination (no change in colour and no fibrillation). For chondrocyte isolation, aseptically dissected cartilage was subjected to sequential digestion with pronase (catalogue number 165921; Roche Molecular Biochemicals, Indianapolis, IN, USA) and collagenase P (catalogue number 1213873; Roche Molecular Biochemicals) at a final concentration of 1 mg/ml in Dulbecco's modified Eagle's medium/F12 plus 10% foetal calf serum and sterilized by filtration, in accordance with the manufacturer's instructions. In our hands, this procedure was superior to enzymatic isolation with collagenase alone in terms of chondrocyte yields and capacity for attachment. Cartilage specimens were finely diced in phosphate-buffered saline (PBS), and after removing PBS diced tissue was incubated for 30 min with pronase in a shaking water bath at 37°C. Pronase was subsequently removed from the digestion flask and the cartilage pieces were washed with PBS. After removal of PBS, digestion was continued with addition of collagenase P; this was done over 6–8 hours in a shaking water bath at 37°C. The resulting cell suspension was filtered through a 40 μm nylon cell strainer (BD Biosciences Europe, Erembodegem, Belgium) in order to remove debris. Cells were centrifuged and washed twice with PBS, counted and plated in 24-well tissue culture plates for chondrocyte culture. Cells were serially passaged to obtain a sufficient number of cells and used between the first and second passages.

### Cell treatments and nitrite assay

ATDC5 cells and human primary chondrocytes, with a viability greater than 95% as evaluated using the trypan blue exclusion method, were cultured (as described above) in 24-well plates. After 12 hours of starvation in serum-free medium, cells were stimulated for 48 hours with leptin (800 nmol/l), alone or in combination with IL-1 (0.025 ng/ml). We wished to determine whether increased NO production was due to NOS type II activation and to the involvement of JAK2, phosphatidylinositol 3-kinase (PI3K), mitogen-activated protein kinase kinase (MEK)1 and p38 kinase. For this purpose, the following specific pharmacological inhibitors were added 1 hour before cytokine stimulation: aminoguanidine (1 mmol/l) for NOS type II; tyrphostin AG490 (5 and 10 μmol/l) and Tkip (20 and 50 μmol/l) for JAK2; wortmannin (1, 2.5, 5 and 10 μmol/l) and LY294002 (1, 2.5, 5 and 10 μmol/l) for PI3K; PD098059 (1, 5, 10, 20 and 30 μmol/l) for MEK-1; and SB203580 (1, 5, 10, 20 and 30 μmol/l) for p38 kinase. Cytokines and pharmacological inhibitor doses were selected on the basis of prior dose–response experiments (data not shown) or previously published literature [15].

Nitrite accumulation was measured in culture medium using the Griess reaction. Briefly, 100 μl cell culture medium was mixed with 100 μl Griess reagent (equal volumes of 1% [weight/vol] sulfanilamide in 5% [vol/vol] phosphoric acid and 0.1% [weight/vol] naphtylethylenediamine-HCl), incubated at room temperature for 10 min, and then the absorbance at 550 nm was measured using a microplate reader (Titertek-Multiscan, Labsystem, Helsinki, Finland). Fresh culture medium was used as blank in all of the experiments. The amount of nitrite in the samples (in micromolar units) was calculated from a sodium nitrite standard curve freshly prepared in culture medium.

### RNA isolation and real-time RT-PCR

ATDC5 chondrogenic cells were seeded in P6 well plates to reach 85–90% confluence. After 8 hours of starvation in serum-free medium, cells were treated with leptin alone or in combination with IL-1. In order to test the involvement of JAK2, PI3K, MEK-1 and p38 kinase on NOS type II mRNA expression, specific inhibitors (tyrphostin AG490 10 μmol/l, wortmannin and LY294002 10 μmol/l, PD098059 30 μmol/l and SB203580 30 μmol/l) were added 1 hour before cytokine stimulation. After 48 hours of treatment, RNA was isolated from cell culture using the Trizol-LS^®^TM method (Gibco-BRL, Life Technologies, Grand Island, NY USA), in accordance with the manufacturer's instructions. Briefly, 5 × 10^5 ^cells were lysed in 1000 μl Trizol-LS^® ^reagent, and recovery of total RNA after isopropanol precipitation was measured using a spectrophotometer (Beckman DU62, Amersham Biosciences, Chalfont St. Giles, UK) at 260 nm.

### Analysis of nitric oxide synthase type II gene expression using real-time RT-PCR

Real-time RT-PCR analyses were performed in a fluorescent temperature cycler (MX3000P Real Time PCR System; Stratagene), in accordance with the manufacturer's instructions. Total RNA 1 μg was used for each RT reaction. cDNAs were synthesized using 200 units of Moloney murine leukaemia reverse transcriptase (Gibco-BRL) and 6 μl dNTPs mix (10 mmol/l of each dNTP), 6 μl of first strand buffer (250 mmol/l Tris-HCl [pH 8.3], 375 mmol/l KCl, 15 mmol/l MgCl_2_; Gibco-BRL), 1.5 μl of 50 mmol/l MgCl_2_, 0.17 μl random hexamer solution (3 μg/μl; Gibco-BRL) and 0.25 μl of RNAse OutTM (recombinant ribonuclease inhibitor 40 μg/μl; Gibco-BRL), in a total volume of 30 μl. Reaction mixtures were incubated at 37°C for 50 min and at 42°C for 15 min. The RT reaction was terminated by heating at 95°C for 5 min and subsequently quick chilled on ice. The 50 μl amplification mixture (Brilliant SYBR Green QPC Master Mix; Stratagene) contained 2 μl of RT reaction products plus 0.75 μl (30 nmol/l) diluted reference dye, 150 nmol/l of each primer and nuclease-free, PCR grade water to adjust the final volume to 50 μl.

After a first enzyme activation step (95°C for 10 min), reactions were cycled 33 times using the following parameters for NOS type II detection: denaturation at 95°C for 40 s, annealing at 60°C for 1 min and extension at 72°C for 1 min. Mouse glyceraldehyde-3-phosphate dehydrogenase (GAPDH) cDNA (5'-TCCATGACAACTTTGGCATCGTGG-3' for upstream primer and 5'-GTTGCTGTTGAAGTCACAGGAGAC-3' for downstream primer; Genebank M32599) was amplified under the same conditions and was used as a normalizer gene. The amount of PCR products formed in each cycle was evaluated on the basis of SYBR Green I fluorescence. A final extension at 72°C over 10 min was followed by melting curve profiles as follows: 95°C for 1 min, ramping down to 45°C at a rate of 0.2°C/s, and heating slowly (0.5°C/cycle) to 95°C for a total of 81 cycles (30 s/cycle). Fluorescence was measured continuously to confirm amplification of specific transcripts (data not shown).

The oligonucleotide primers specific for mouse NOS type II were as follows: upstream primer 5'-CTCACTGGGACAGCACAGAA-3' and downstream primer 5'-TGGTCAAACTCTTGGGGTTC-3' (from Genbank U43428).

Cycle-to-cycle fluorescence emission readings were monitored and quantified using the second derivative maximum method from the MX3000P Real Time software package (Stratagene). This method determines the crossing points of individual samples using an algorithm that identifies the first turning point of the fluorescence curve. This turning point corresponds to the first maximum of the second derivative curve and correlates inversely with the log of the initial template concentration. NOS type II mRNA levels were normalized with respect to mouse GAPDH level in each sample.

### Nitric oxide synthase type II western blot analysis

ATDC-5 chondrogenic cells were seeded in P100 plates until they reached 85–90% confluence. After overnight starvation in serum-free medium, cells were stimulated for 24 hours with leptin (800 nmol/l), alone or in combination with IL-1 (0.025 ng/ml). In order to demonstrate the involvement of JAK2, PI3K, MEK-1 and p38 kinase, the following specific pharmacological inhibitors were added 1 hour before cytokine stimulation: tyrphostin AG490 (5 and 10 μmol/l) and Tkip (20 and 50 μmol/l) for JAK2; LY294002 (1, 5 and 10 μmol/l) for PI3K; PD098059 (1, 10 and 30 μmol/l) for MEK-1; and SB203580 (1, 10 and 30 μmol/l) for p38 kinase. After stimulation, cells were rapidly washed with ice cold PBS and scraped in lysis buffer: 10 mmol/l Tris-HCl (pH 7.5), 5 mmol/l EDTA, 150 mmol/l NaCl, 30 mmol/l sodium pyrophosphate, 50 mmol/l sodium fluoride, 1 mmol/l sodium orthovanadate (Na_3_VO_4_), 10% glycerol, 0.5% Triton X-100, 1 mmol/l phenylmethylsulfonilfluoride, aprotinin, leupeptin and pepstatin A (10 mg/ml). Lysed cells were centrifuged at 13000 *g *for 15 min. Lysates from control or stimulated cells were collected and separated by SDS-PAGE on a 10% polyacrylamide gel. Proteins were subsequently transferred to a polyvinylidene difluoride transfer membrane (Hybond TM-P; Amersham International, Little Chalfont, UK) using a transfer semidry blot cell (BioRad Laboratories, Hercules, CA, USA). Blots were incubated with the appropriate antibody (mouse anti-NOS II antibody; purchased from Upstate Biotech, Lake Placid, NY, USA). Immunoblots were visualized using ECLPlus detection Kit (Amersham-Pharmacia Biotech, Barcelona, Spain) using horseradish peroxidase labelled secondary antibody. To confirm equal load in each sample, after stripping in glycine buffer at pH 3, membranes were reblotted with anti-actin antibody (Santa Cruz Biotechnology Inc., Santa Cruz, CA, USA). The images of autoradiograms were captured and analyzed using a Typhoon 9410 digital variable mode imager (Amersham Biotech, Little Chalfont, UK).

### Data analysis

Data are expressed as mean ± standard error of the mean of at least three independent experiments, each with at least three or more independent observations. Statistical analysis was performed using analysis of variance followed by the Student–Newman–Keuls or Bonferroni multiple comparison test with the Instat computerized package (GraphPad Software Inc., San Diego, CA, USA). i < 0.05 was considered statistically significant.

## Results

### Leptin synergistic effect over IL-1 induced nitrite production in chondrocytes

**Figure 1:**
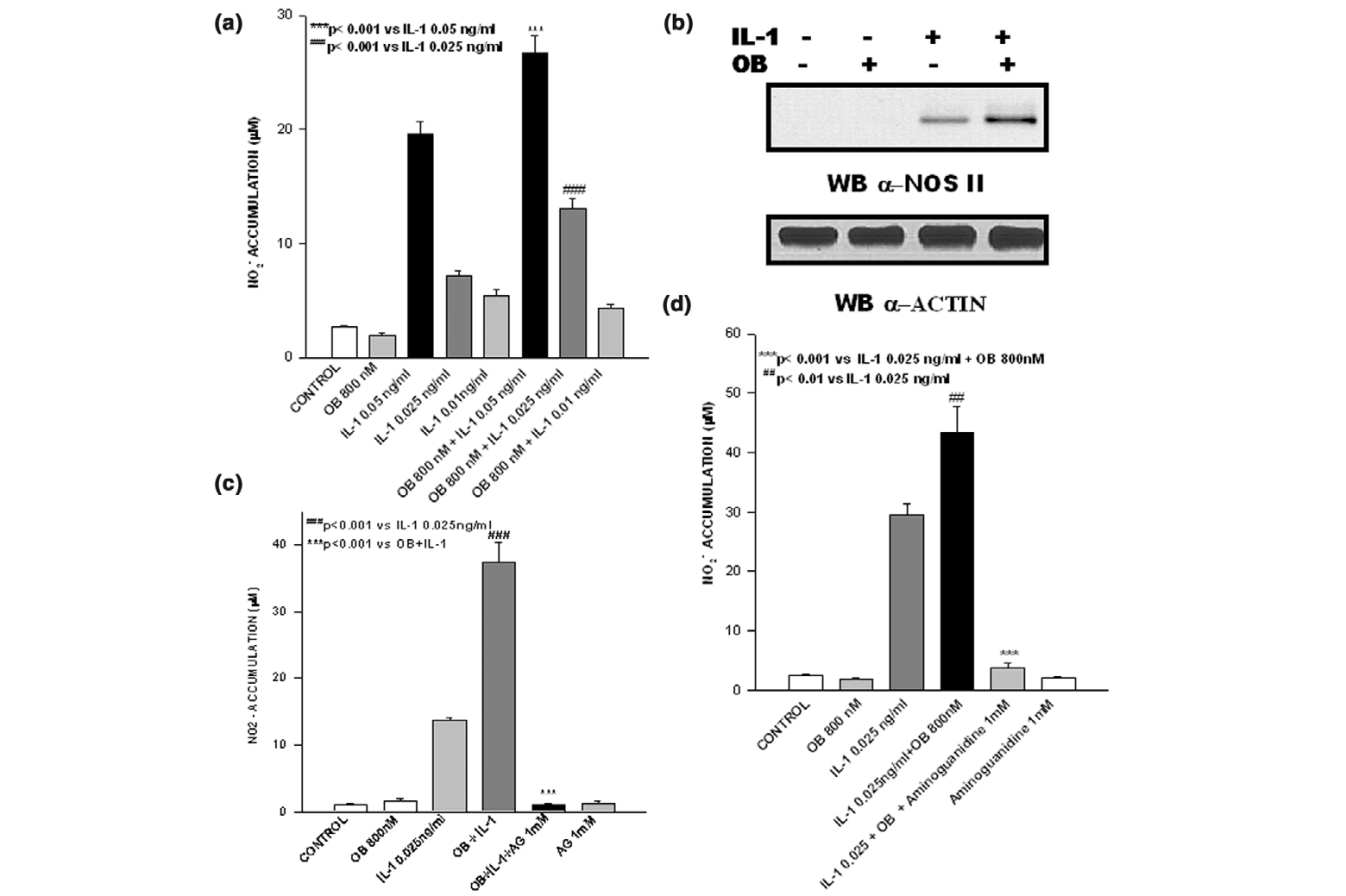
Leptin synergizes with IL-1 in inducing nitric oxide synthase (NOS) type II. Synergistic effect of leptin (OB) on nitrite (NO_2_^-^) accumulation and NOS type II protein expression induced by IL-1. Stimulations were conducted in serum-free conditions **(a,b) **in ATDC5 chondrogenic cells and **(c) **in human primary chondrocytes. NO_2_^- ^accumulation is selectively inhibited by aminoguanidine (AG) both in **(d) **ATDC5 cells and in (panel c) human primary chondrocytes. Values are expressed as mean ± standard error of the mean. WB, western blot.

A leptin concentration of 800 nmol/l was found to be optimal for co-stimulatory experiments. This concentration was selected based on a braod set of previous dose–response experiments (data not shown). Because NOS type II stimulation with IL-1 at 0.05 ng/ml was maximal, a dose of 0.025 ng/ml was selected in order to avoid masking leptin synergism. As shown in Fig. 1, ATDC5 cells and human primary chondrocytes did not accumulate nitrites when stimulated with leptin alone; however, leptin was able to increase significantly nitrite accumulation induced by IL-1 when cells were co-stimulated with both cytokines (Fig 1a,c). This result was confirmed in terms of protein expression. Indeed, a clear-cut increase in levels of NOS type II protein was observed when cells were co-stimulated with leptin and IL-1 (Fig. 1b).

To confirm whether NO formation was produced via NOS type II, ATDC5 cells and human chondrocytes were incubated for 48 hours with both cytokines in the presence of the NOS type II inhibitor aminoguanidine (1 mmol/l), added 1 hour before cytokine administration. Aminoguanidine completely inhibited nitrite accumulation in the culture supernatant of human primary chondrocytes (Fig. 1c) and ATDC5 cells (Fig. 1d).

### Janus kinase-2 inhibition blocks leptin/IL-1 induced nitric oxide production and nitric oxide synthase type II protein expression

**Figure 2:**
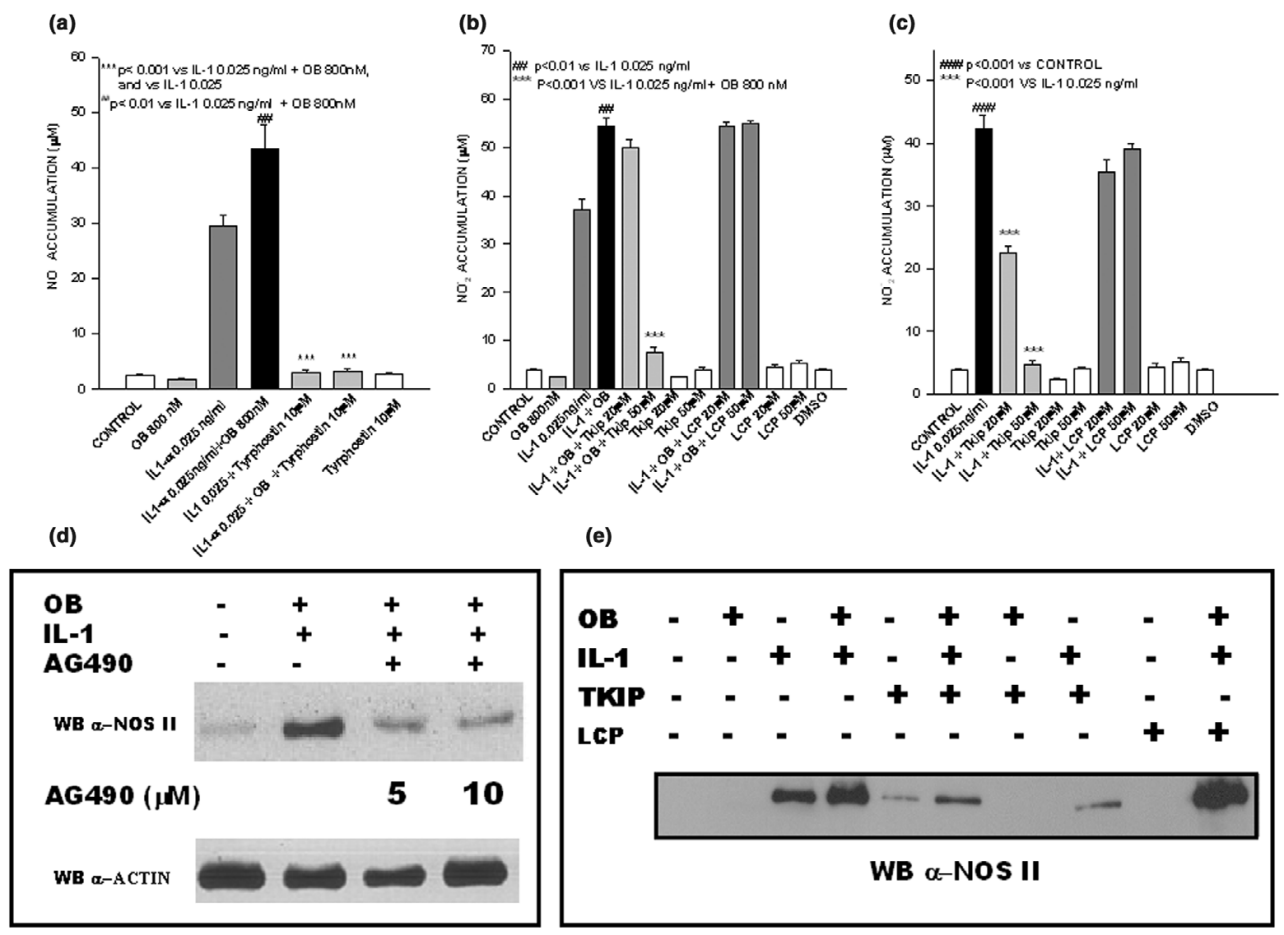
Janus kinase (JAK)2 inhibition blocks leptin/IL-1-induced nitric oxide (NO) production and nitric oxide synthase (NOS) type II protein expression. Effect of tyrphostin AG490 and Tkip on NO production and NOS II protein expression. The effect of tyrphostin AG490 was evaluated in terms of **(a) **nitrite accumulation in ATDC5 cells stimulated with leptin and IL-1, and in terms of **(d) **NOS II protein expression. The effect of Tkip was evaluated by nitrite accumulation in **(b) **leptin/IL-1 ATDC5 co-stimulated cells and in **(c) **IL-1 stimulated cells (panel c). **(e) **Effect of Tkip on NOS type II protein expression in leptin/IL-1 co-stimulated cells.

We also investigated the role played by JAK2 in nitrite production evoked by co-stimulation with leptin and IL-1 by using tyrphostin AG490. This JAK2 inhibitor, added 1 hour before cytokine co-stimulation, completely blocked nitrite production (Fig. 2a). This result was confirmed in terms of protein expression, because cell pretreatment with tyrphostin AG490 significantly decreased NOS II protein expression in leptin/IL-1 co-stimulated cells (Fig. 2d). Intriguingly, tyrphostin AG490 was also able to inhibit nitrite accumulation induced by IL-1 alone, suggesting that leptin synergizes with fundamental pathways in IL-1 responses. To gain further insights into the involvement of JAK2, Tkip (a 12-mer SOCS-1 mimetic peptide that binds to the autophosphorylation site of JAK2) was added to ATDC5 cells 1 hour before they were stimulated with leptin or IL-1, or both cytokines. Tkip at 50 μmol/l was able to blunt completely leptin/IL-1 induced nitrite accumulation and NOS II protein expression (Fig. 2b,e). A lipophilic irrelevant peptide, MuIFN-γ_95–125 _(AKFEVNNPQVQRQAFNELIRVVHQLLPESSL), was used as control. Intriguingly, Tkip was also able to inhibit, in a dose–response manner, nitrite accumulation and NOS II protein expression in ATDC5 cells stimulated with IL-1 alone (Fig. 2c,e).

### Effect of the specific signalling pathways inhibitors LY294002, PD098059 and SB203580 on leptin/IL-1 co-stimulation

In order to define the signalling pathway involved in the synergistic induction of NOS type II mediated by co-stimulation with leptin and IL-1 in cultured ATDC5 cells, we evaluated the effects of specific pharmacological inhibitors on other kinases, specifically PI3K, MEK-1 and p38 kinase.

**Figure 3:**
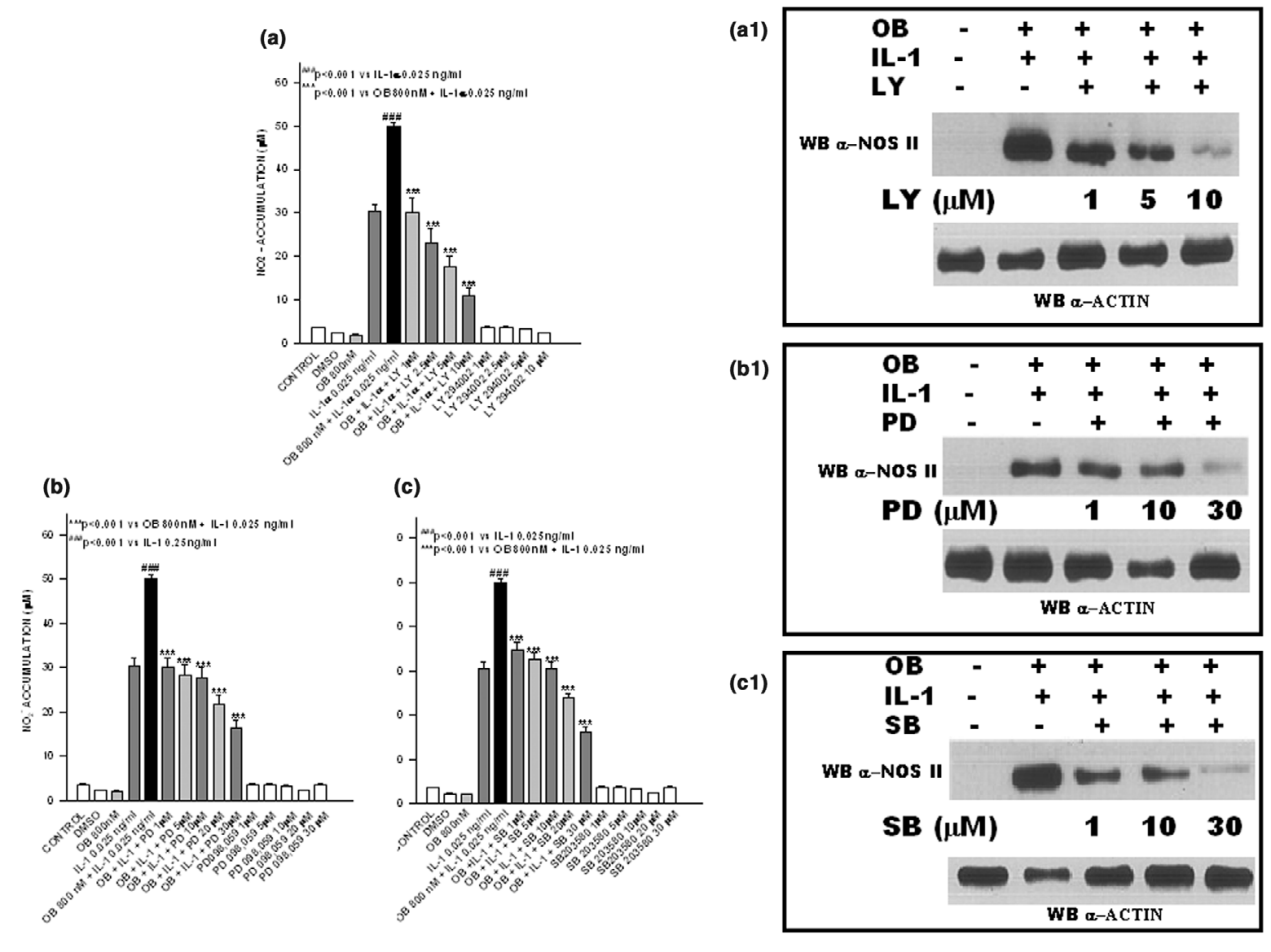
Involvement of phosphatidylinositol 3-kinase (PI3K), mitogen-activated protein kinase kinase (MEK)-1 and p38-kinase in leptin/IL-1-induced nitric oxide synthase (NOS). Dose-dependent effect of **(a,a1) **LY294002, **(b,b1) **PD098059 and **(c,c1) **SB203580 on nitrite (NO_2_^-^) production and NOS type II protein expression in stimulated and unstimulated ATDC5 cells. Stimulations were conducted in serum-free conditions. Each inhibitor was added 1 hour before cytokine co-stimulation. Values are expressed as mean ± standard error of the mean. OB, leptin; WB, western blot.

We first investigated the effect of a specific inhibitor of PI3K, namely LY294002 (1, 2.5, 5 and 10 μmol/l) on leptin/IL-1 induced NO production. The addition of LY294002 1 hour before cytokine co-stimulation resulted in significant and dose-dependent decreases in NO production and NOS type II protein expression (Fig. 3a,a1).

In order to test whether MEK-1 (the mitogen-activated protein kinase [MAPK] kinase involved in extracellular signal-regulated kinase [ERK]-1 and ERK-2 phosphorylation/activation) participates in NOS type II induction via leptin/IL-1 co-stimulation, we used the specific MEK-1 inhibitor PD98059. When this inhibitor was added 1 hour before cytokine co-stimulation, significant dose-dependent decreases in NO production and NOS II protein expression were observed (Fig. 3b,b1).

Finally, because it has been shown that p38 kinase is involved in apoptotic processes induced by NO in chondrocytes, we tested whether this MAPK is also involved in NOS type II synergistic activation stimulated by leptin/IL-1. For this purpose, we used the specific p38 kinase inhibitor SB203580. Addition of this inhibitor 1 hour before leptin/IL-1 co-stimulation caused significant and dose-dependent decreases in NO production and NOS II protein expression (Fig. 3c,c1 [lower panel]).

### Leptin synergism does not depend on chondrocyte differentiation state

**Figure 4:**
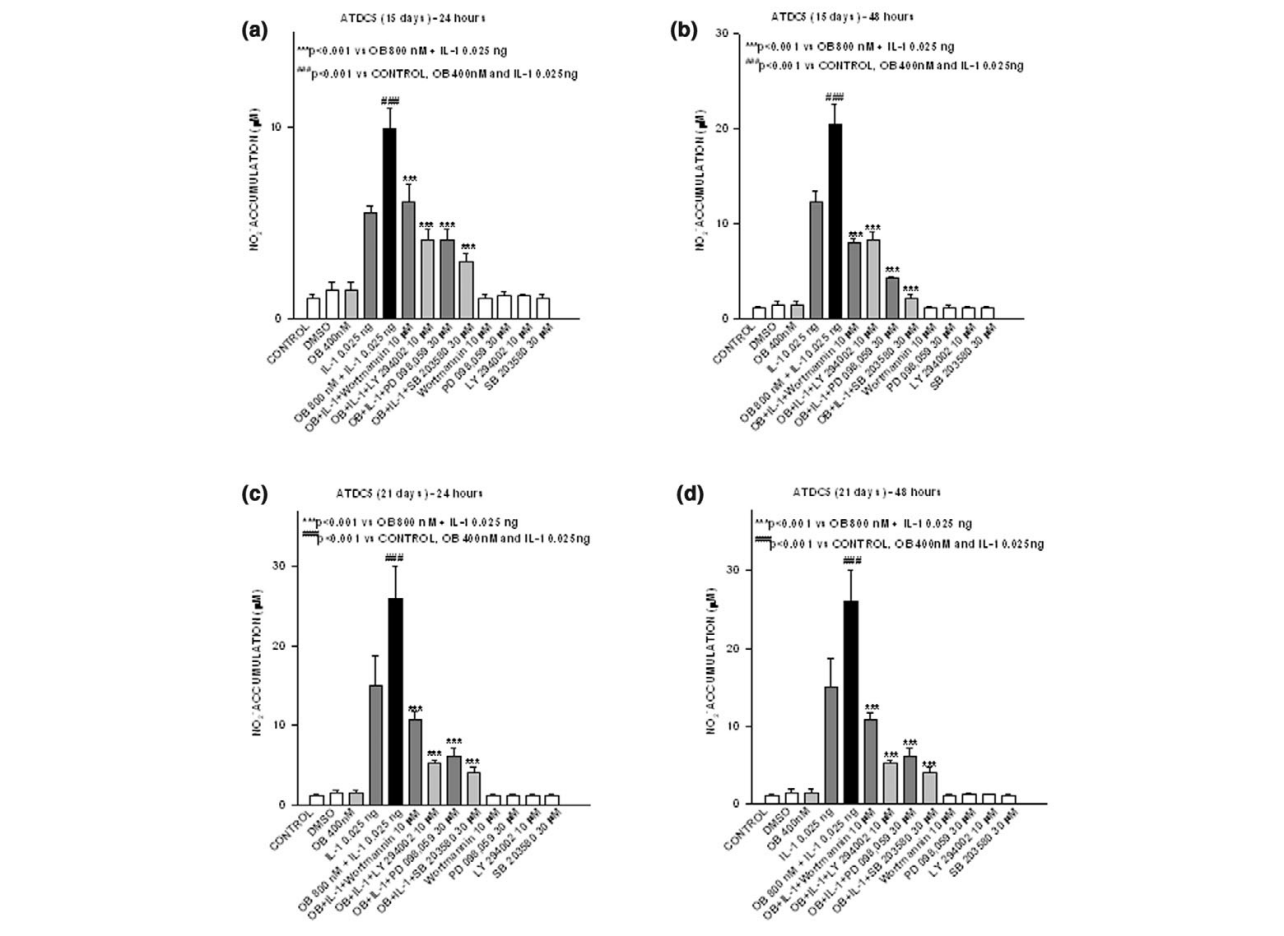
Leptin synergism does not depend upon chondrocyte differation state. Effect of different inhibitors on nitrite (NO_2_^-^) accumulation in 15-day differentiated ATDC5 cells stimulated or not with leptin, alone or in combination with IL-1, during **(a) **24 and **(b) **48 hours. The effect of inhibitors was also evaluated in 21-day differentiated ATDC5 cells, after **(c) **24 or **(d) **48 hours of stimulation with leptin and IL-1 (alone or in combination). Values are expressed as mean ± standard error of the mean. OB, leptin.

In order to determine whether leptin/IL-1 synergism and its signalling pathway depend on the differentiation state of chondrocytes, we conducted similar experiments in mature and hypertrophic chondrocytes. We differentiated ATDC5 cells (see Materials and methods, above) into mature and hypertrophic chondrocytes, and tested co-stimulation and treatments with all specific inhibitors. Nitrite accumulation, evaluated in 15-day (mature) and in 21-day (hypertrophic) differentiated ATDC5 cells at 24 and 48 hours after treatment, was similar to that observed in the ATDC5 chondrogenic undifferentiated cell line (Fig. 4a–d). Note that in order to evaluate the involvement of PI3K, in some experiments we also used wortmannin at 10 μmol/l (a classical but not very specific PI3K inhibitor), yielding results similar to those obtained with LY294002.

**Figure 5:**
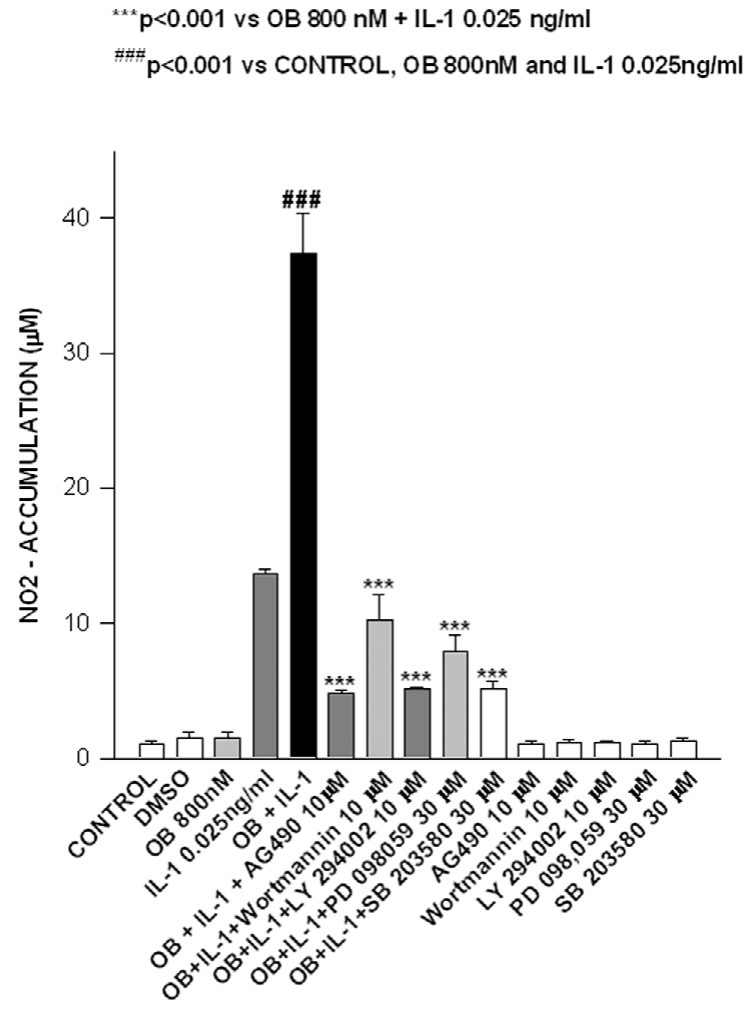
Leptin acts synergistically with IL-1 in human primary chondrocytes. Nitrite (NO_2_^-^) accumulation in leptin (OB)/IL-1 co-stimulated human primary chondrocytes. Stimulations were conducted in serum-free conditions in the presence or absence of tyrphostin AG490, wortmannin, LY294002, PD98059 and SB203580 inhibitors. Values are expressed as mean ± standard error of the mean.

Finally, a similar pattern was observed in human cultured primary chondrocytes. In these cells, leptin induced a strong increase in nitrite accumulation over that induced by IL-1, and the synergistic response was significantly inhibited by tyrphostin AG490, wortmannin, LY294002, PD98059 and SB203580 (Fig. 5).

### Effect of leptin/IL-1 co-stimulation on nitric oxide synthase type II RNA expression

**Figure 6:**
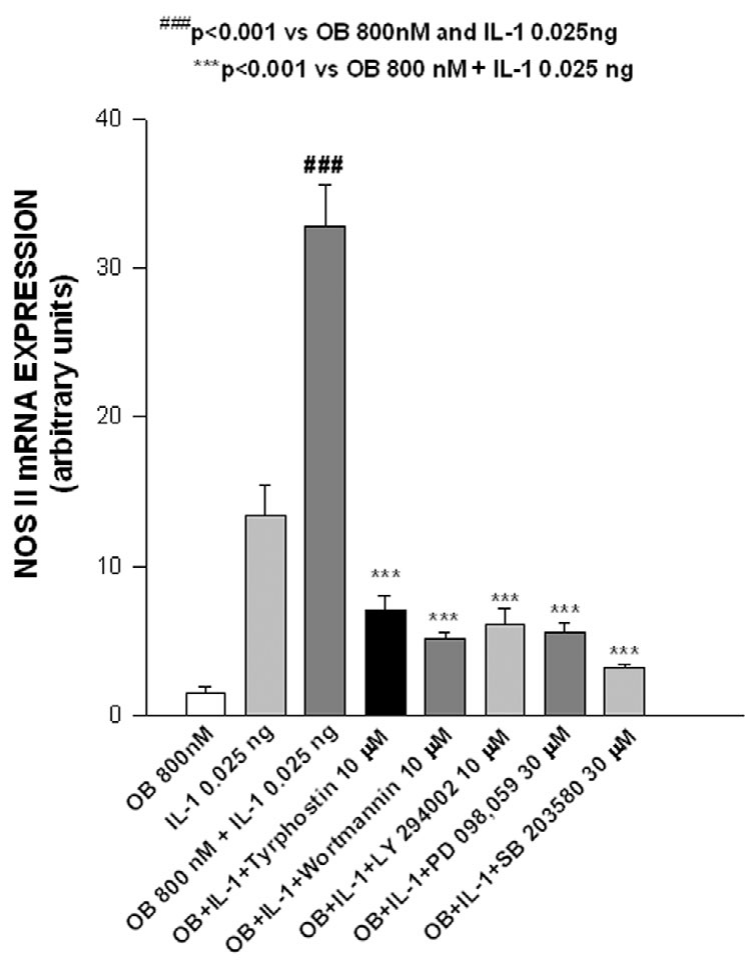
Effect of leptin/IL-1 co-stimulation on nitric oxide synthase (NOS) type II mRNA expression. Real-time RT-PCR analysis of the expression of the inducible NOS type II mRNA in leptin (OB)/IL-1 co-stimulated ATDC5 cells. Stimulations (24 hours) were conducted in serum-free conditions. Specific inhibitors were added 1 hour before cytokine co-stimulation. Values are expressed as mean ± standard error of the mean.

We finally studied NOS II mRNA expression in order to determine whether NO increase/inhibition was due to modulation of NOS type II mRNA expression. As shown in Fig. 6, NOS type II mRNA, evaluated using real-time PCR, was strongly expressed when cells were co-stimulated with leptin plus IL-1, and this expression was significantly reduced by tyrphostin AG490, wortmannin, LY294002, PD098059 and SB203580.

## Discussion

In the present study we investigated the effect of leptin on NO production stimulated by IL-1. We found that leptin had a synergistic effect in the ATDC5 murine chondrogenic cell line, in differentiated mature and hypertrophic ATDC5 chondrocytes, and in human primary chondrocytes.

Leptin has been classified as a cytokine-like hormone, because of its structure and the homology of its receptors with members of the class I cytokine receptor superfamily. A proinflammatory role for leptin has previously been proposed. Several data show that leptin levels are increased by proinflammatory cytokine administration and in animal models of acute inflammation [9]. In addition, leptin regulates not only humoral but also cellular immune responses in antigen-induced arthritis models [20]. Nevertheless, there are only few reports of a direct action of leptin at the cellular level in cartilage [14,15].

NO controls a variety of cartilage functions, including loss of chondrocyte phenotype, chondrocyte apoptosis, and extracellular matrix degradation [2,3]. NOS type II is mainly expressed by immune cells in response to a wide range of proinflammatory cytokines [21,22]. *In vitro*, human articular cartilage is able to produce large amounts of NO [23], which can be enhanced by proinflammatory cytokines. In addition, NO production can be significantly increased by the presence of leptin, as shown in our previous work [15] and in the present study.

Here, we show that the IL-1 induced production of NO by ATDC5 murine chondrocytes and by human chondrocytes is significantly enhanced by leptin. It is noteworthy that, apart from blood, several sources of leptin and IL-1 have been identified in or around the joints in pathological conditions. IL-1 is produced by inflamed synovium and periarticular fat pad [24]. Interestingly, multipotent stromal cells from the infrapatellar fat produce leptin [25]. In addition, osteoarthritic human chondrocytes produce leptin, and leptin administration in rats induces over-expression of this hormone by articular chondrocytes [13]. Thus, in patients with inflammatory synovitis or osteoarthritis, there is a unique microenvironment in the cartilage characterized by elevated levels of both leptin and IL-1, due not only to local production but also to systemic increase [10,13,26]. It is conceivable that in this scenario leptin plays a significant proinflammatory role, as suggested by the findings presented here. Of further interest is our previous report [15] of the co-stimulatory effect of leptin and IFN-γ at the chondrocyte level.

We previously established that the early event in leptin/IFN-γ synergistic NOS type II activation was the involvement of JAK2 [15]; the present results confirm that JAK2 activation is also an early step in leptin/IL-1 induced NOS type II co-stimulation. The fact that tyrphostin AG490 blocks the leptin/IL-1 response implies that leptin synergizes with critical pathways in IL-1 response. It was surprising that tyrphostin AG490 also blocked the response to IL-1 alone, because JAK2 is not known to be required for IL-1 receptor transduction, and so one would expect the effect of tyrphostin AG490 to be partial. However, our results are in agreement with those reported by other investigators [27,28].

We also used Tkip in our experiments; Tkip is a 12-mer SOCS-1 mimetic lipophilic peptide (WLVFFVIFYFFR) that inhibits JAK2 autophosphorylation [29]. Interestingly, the behaviour of this peptide was similar to that of tyrphostin AG490 in terms of NOS II inhibition. It is conceivable that this peptide, because of its SOCS-1 mimetic properties, could inhibit IL-1/Toll-like receptor function in chondrocytes. SOCS-1 is a negative regulator of lipopolysaccharide-induced macrophage activation [30,31] and has been shown to bind to IL-1 receptor associated kinase [32]. This disrupts the cascade that leads to nuclear factor-κB (NF-κB) signalling and causes NOS inhibition. Of note, it has been demonstrated that tyrphostin AG490 inhibits IL-1 induced NF-κB activation in concentrations that also inhibit NOS II mRNA and protein synthesis. These findings suggest that JAK2 is required for NF-κB activation, which in turn mediates IL-1 induced NOS II expression in chondrocytes [28].

To gain further insights into the mechanism by which leptin, together with IL-1, promotes NO production, we evaluated the roles played by downstream signalling cascades using specific pharmacological inhibitors. First, we analyzed the involvement of PI3K. The role played by this kinase in the activation of NOS type II is quite controversial and remains the subject of debate. A number of studies support the view that PI3K activity down-regulates NOS type II, but there are several caveats to this view. For instance, insulin-like growth factor-II stimulates NOS type II expression and activity in myoblasts via a PI3K-dependent mechanism involving IκBα degradation and increased p65 NF-κB DNA binding activity [33], which is in agreement with recent evidence indicating that PI3K/protein kinase B is involved in NF-κB activation [34,35]. In addition, PI3K homologues (mammalian target of rapamycin/FKBP12–rapamycin associated protein) have been implicated in the phosphorylation and activation of NOS type II [36]. It should therefore be stressed that the interaction between NOS type II and PI3K may vary depending on the cell model, and so this interaction may be subject to tissue-specific regulation.

Our results also suggest that ERK-1/2 and p38 kinase play pivotal roles in the activation of NOS type II mediated by leptin/IL-1 co-stimulation. We found that ERK-1/2-specific pharmacological inhibition significantly decreased NO production induced by leptin/IL-1 co-stimulation in cultured chondrocytes. This result is in agreement with previous studies that associated ERK-1/2 activation with NOS type II induction by a combination of proinflammatory stimuli [37-40].

Finally, we found that the blockade of p38 kinase caused a significant decrease in NO production, NOS II mRNA expression and NOS II protein level. These data are concordant with previous reports that implicate p38 kinase in NOS type II upregulation in macrophages [41], neural cells [42,43] and chondrocytes [44].

Synergistic interactions of IL-1 with other soluble factors are not novel and have been reported in chondrocytes and other cell types. For instance, IL-1 synergizes with oncostatin M to induce markedly the expression of matrix metalloproteinase (MMP)-1, MMP-3, MMP-8 and MMP-13, as well as aggrecanase ADAM-TS4 [45]. In addition, a synergistic induction of MMP-1 by IL-1 and oncostatin M has been observed in human chondrocytes via a novel mechanism that involves STAT (signal transducer and activator of transcription) and activator protein-1 [46].

As far as we are aware, this is the first report that demonstrates the cooperative interaction between leptin and IL-1 in the induction of NO production in activated chondrocytes.

## Conclusion

The present study shows that in human and ATDC5 murine cultured chondrocytes, leptin, together with IL-1, significantly increases the production of NO by a mechanism that implies upregulation of NOS type II mRNA and protein. In this modulation, an intracellular signalling pathway that involves JAK2 tyrosine kinase, PI3K and two members or the MAPK pathway (ERK and p38) is at play. The functional interplay of these pathways may be important for the onset as well as the maintenance of inflammatory responses in cartilage.

## Abbreviations

ERK = extracellular signal-regulated kinase; GAPDH = glyceraldehyde-3-phosphate dehydrogenase; IFN = interferon; IL = interleukin; JAK = janus kinase; MAPK = mitogen-activated protein kinase; MEK = mitogen-activated protein kinase kinase; MMP = matrix metalloproteinase; NF-κB = nuclear factor-κB; NO = nitric oxide; NOS = nitric oxide synthase; PBS = phosphate-buffered saline; PI3K = phosphatidylinositol 3-kinase; RT-PCR = reverse transcription polymerase chain reaction; SOCS = suppressor of cytokine signalling.

## Competing interests

The author(s) declare that they have no competing interests.
